# Supplementary material for: Phage therapy minimally affects the water microbiota in an Atlantic salmon (Salmo salar) rearing system while still preventing infection
Source: Sci Rep. 2023 Nov 6;13:19145. doi: 10.1038/s41598-023-44987-7 (PMC10628140; doi:10.1038/s41598-023-44987-7)
Supplement: Supplementary file 1 — Supplementary Figures. [file 41598_2023_44987_MOESM1_ESM.pdf]

**Supplementary Materials:**

# Phage therapy minimally affects the water microbiota in an Atlantic salmon (*Salmo salar*) rearing system while still preventing infection

Alexander W. Fiedler[1], Madeleine S. Gundersen[1], Toan P. Vo[1], Eivind Almaas[1], Olav Vadstein[1], Ingrid Bakke[*1]

[1]Norwegian University of Science and Technology, Trondheim, Norway

*Corresponding author: Ingrid Bakke; Department of Biotechnology and Food Science, NTNU; N-7491 Trondheim, Norway; Ingrid.bakke@ntnu.no; Tel. Nr.: +47 73412276

5’-
TTAACACATGCAAGTCGAGGGGTAGAAGGAGCTTGCTCCTTTGAGACCGGCGCACGGGTGCGTAAC
GCGTATGCAATCTACCTTGTACAGGGGGATAGCCCAGAGAAATTTGGATTAATACCCCATAGTATTT
TCAGATGGCCTCATTTGATTATTAAAGTTCCAACGGTACAAGATGAGCATGCGTCCCATTAGCTAGTT
GGTGTGGTAACGGCATACCAAGGCAACGATGGGTAGGGGTCCTGAGAGGGAGATCCCCCACACTG
GTACTGAGACACGGACCAGACTCCTACGGGAGGCAGCAGTGAGGAATATTGGTCAATGGGCGCAA
GCCTGAACCAGCCATGCCGCGTGCAGGATGACGGTCCTATGGATTGTAAACTGCTTTTGTACAGGAA
GAAACCCTCCCTTGTAAGGGAGCTTGACGGTACTGTAAGAATAAGGATCGGCTAACTCCGTGCCAG
CAGCCGCGGTAATACGGAGGATCCAAGCGTTATCCGGAATCATTGGGTTTAAAGGGTCCGTAGGCG
GTTTTATAAGTCAGTGGTGAAATCTGGTCGCTCAACGATCAAACGGCCATTGATACTGTAAGACTTG
AATTACTTGGAAGTAACTAGAATATGTAGTGTAGCGGTGAAATGCTTAGAGATTACATGGAATACCG
ATTGCGAAGGCAGGTTACTACGAGTATATTGACGCTGATGGACGAAAGCGTGGGGAGCGAACAGG
ATTAGATACCCTGGTAGTCCACGCCGTAAACGATGGATACTAGCTGTTTGGAGCAATCTGAGTGGCT
AAGCGAAAGTGATAAGTATCCCACCTGGGGAGTACGCTCGCAAGAGTGAAACTCAAAGGAATTGAC
GGGGGCCCGCACAAGCGGAGGAGCATGTGGTTTAATTCGATGATACGCGAGGAACCTTACCAAGG
CTTAAATGGGAAACGACAGATTTGGAAACAGATCTTTCTTCGGACGTTTTTCAAGGTGCTGCATGGT
TGTCGTCAGCTCGTGCCGTGAGGTGTCAGGTTAAGTCCTATAACGAGCGCAACCCCTGTTGCTAGTT
GCCAGCGAGTCATGTCGGGAACTCTAGCAAGACTGCCGGTGCAAACCGCGAGGAAGGTGGGGATG
ACGTCAAATCATCACGGCCCTTACGCCTTGGGCTACACACGTGCTACAATGGACGGTACAGAGAGCA
GCCACTACGCAAGTAGGAGCGAATCTACAAAACCGTTCTCAGTTCGGATCGGAGTCTGCAACTCGAC
TCCGTGAAGCTGGATTCGCTAGTAATCGCAGATCAGCCATGCTGCGGTGAATACGTTCCCGGGCCTT
GTACACACCGCCCGTCAAGCCATGGAAGCTGGGGGTACCTGAAGTCGGTGACCGCAAGGAGCTGCC
TTAGGTAAAACTG-3’

Supp. Fig. 1: Partial 16S rRNA sequence of *F. columnare* Fc7. The sequence was obtained by extracting DNA from a pure culture of F. columnare, amplification of the 16S rRNA gene using the primers Eub8F (5’-AGAGTTTGATCMTGGCTCAG-3’) and 1492R (5’-GGTTACCTTGTTACGACTT-3’) and sequencing of the amplicons using Sanger sequencing provided by Eurofins Genomics.

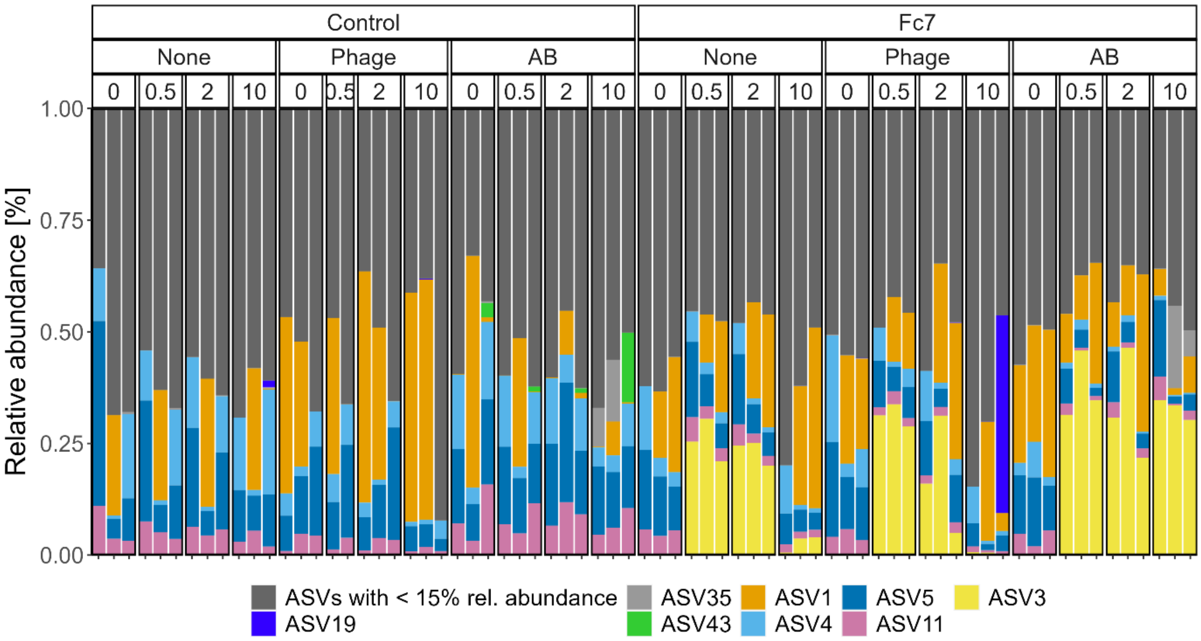


Supp. Fig. 2: Stacked bar graph at ASV level of the bacterial community composition of each water sample taken in Exp.2. All ASVs that do not make up more than 15% of the whole community in at least one sample are summarized as "ASVs with < 15% rel. abundance". The lowest identified taxonomic group for the ASVs shown are: ASV1: *Polaromonas* sp., ASV3: *Flavobacterium columnare* Fc7, ASV4: Comamonadaceae, ASV5: *Janthinobacterium* sp., ASV11: *Janthinobacterium* sp., ASV19: *Pseudomonas* sp., ASV35: Oxalobacteraceae, ASV43: *Flavobacterium* sp.

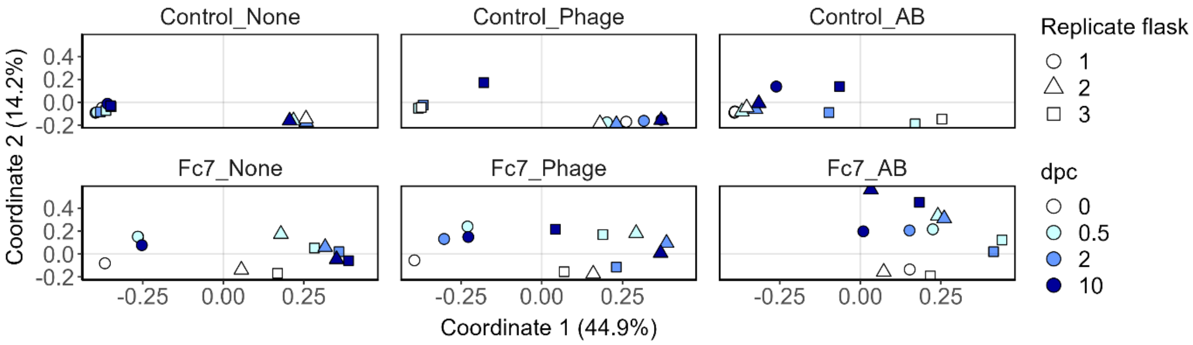


Supp. Fig. 3: PCoA of the Bray-Curtis dissimilarities of all samples from Exp.2. Sampling timepoints are represented by different colours, whereas biological replicate flasks are indicated by different shapes. ASV3 (corresponding to *F. columnare* Fc7) was removed from the dataset prior to generation of the ordination. All panels are from the same PCoA, but were facetted into the different treatment groups.

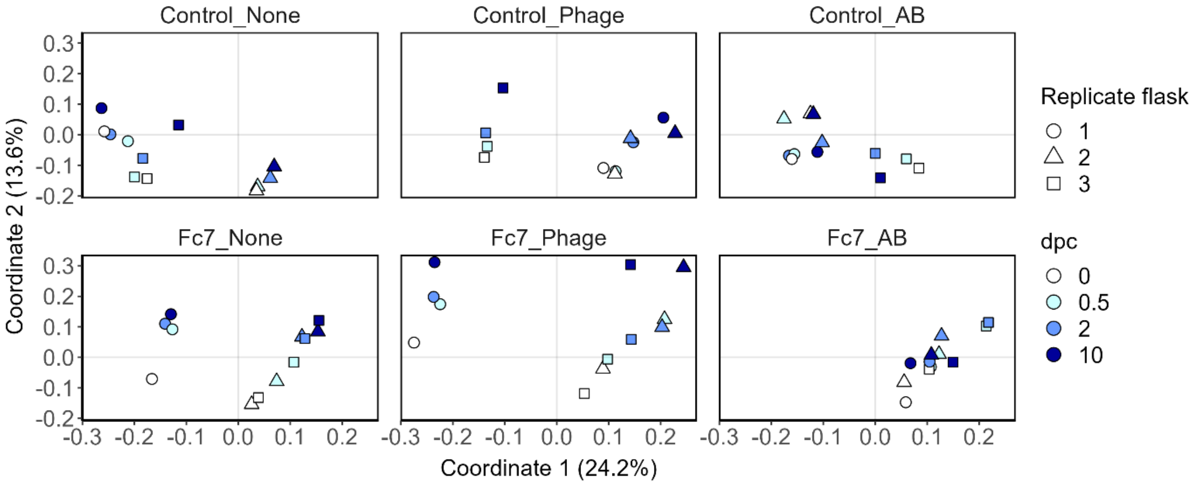


Supp. Fig. 4: PCoA of the Sørensen-Dice indices of the microbiota samples taken in Exp.2. Sampling timepoints are represented by different colours, whereas replicate flasks are indicated by different shapes. Panels are from one PCoA and were facetted into the different treatment groups.

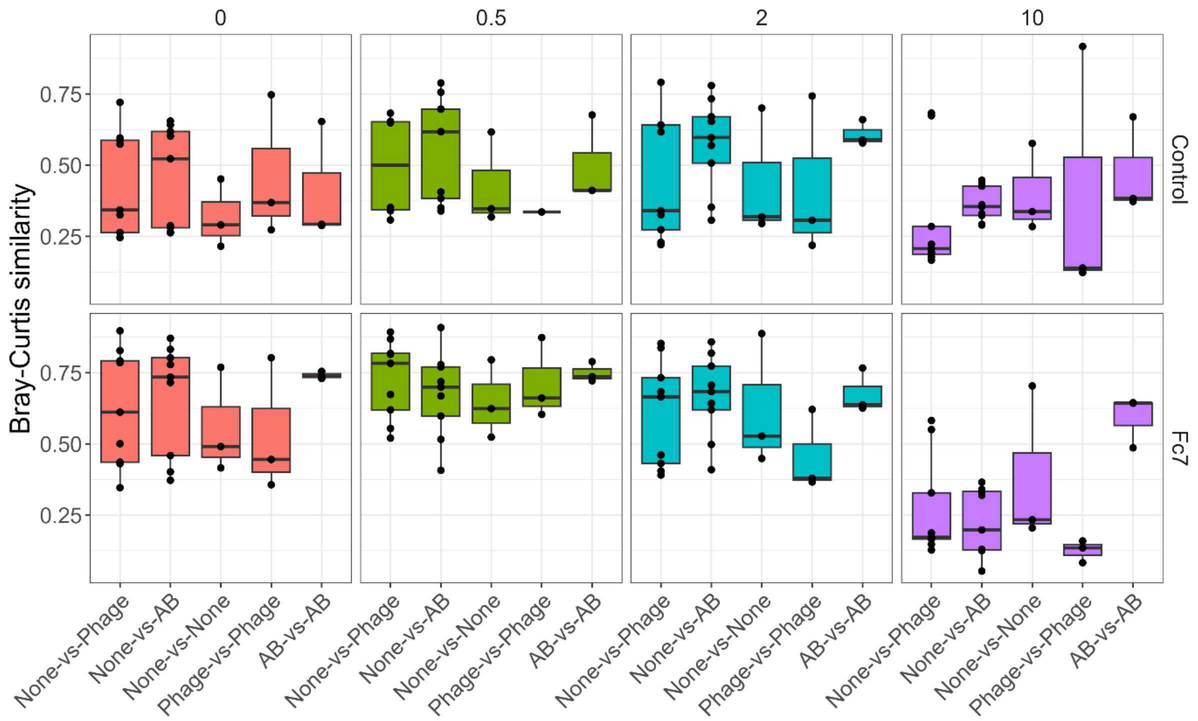


Supp. Fig. 5: Bray-Curtis similarities comparing samples both between and within experimental groups for each timepoint.

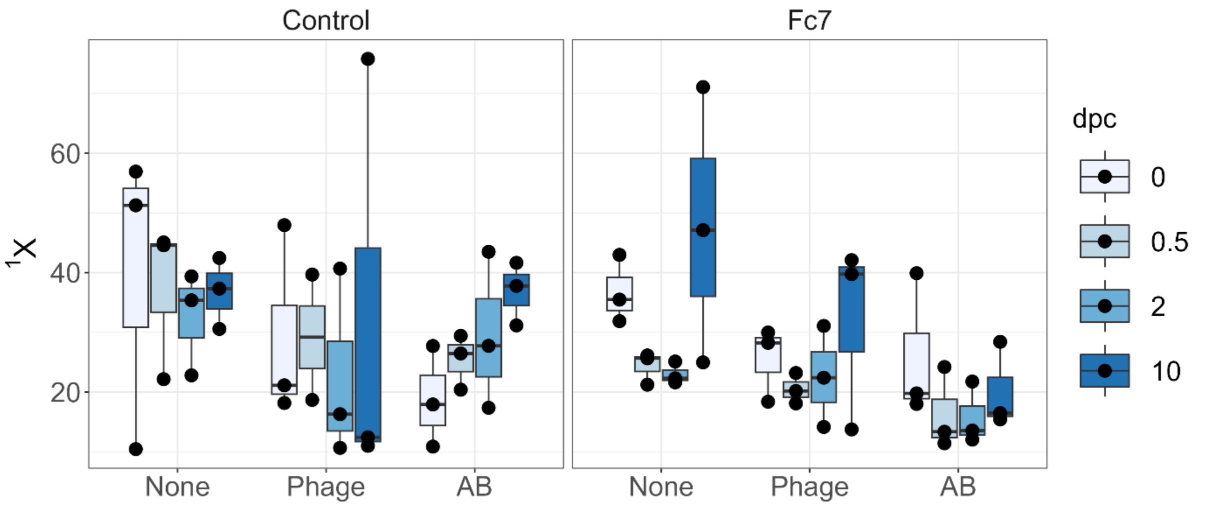


Supp. Fig. 6: α-diversity of all water samples from Exp.2, expressed as Hill's diversity of order 1 (equivalent to the exponential Shannon index). Box plots show median and upper and lower quartile. Whiskers include all samples.

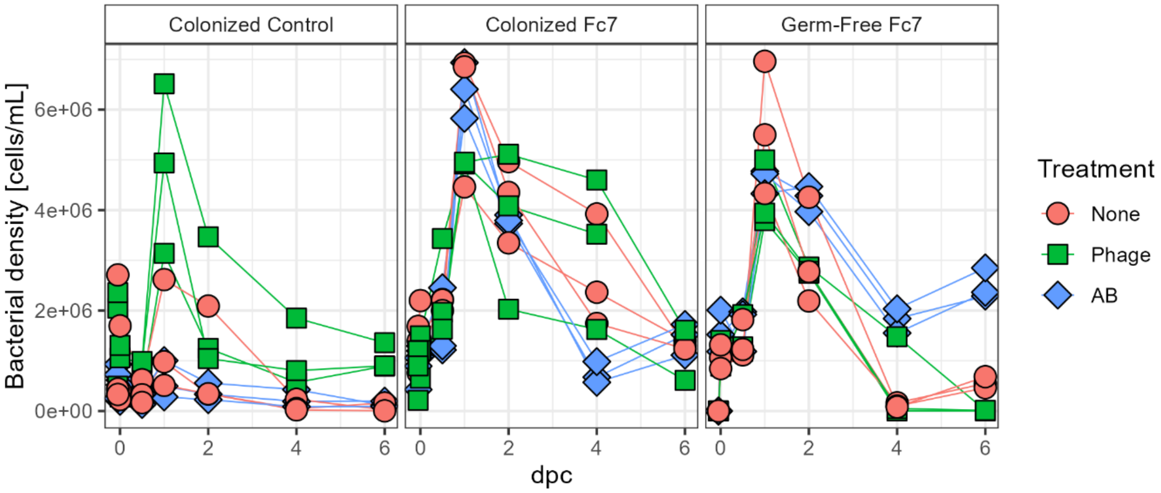


Supp. Fig. 7: The bacterial density [cells per ml] in the rearing water from each flask over time. The samples were taken from the 10 °C experiment and analysed using flow cytometry. At 0 dpc, one sample was taken immediately before and immediately after challenging the flasks with *F. columnare*. At day 1, 4 and 6 the water was exchanged after sampling. Samples from 8 and 10 dpc are not shown due to sample degradation prior to data acquisition. Colours and shapes indicate the treatment type applied at 0 dpc, and the lines connect the bacterial density observations between individual flasks.
